# Comparison of antimicrobial resistant *Escherichia coli* isolated from Irish commercial pig farms with and without zinc oxide and antimicrobial usage

**DOI:** 10.1186/s13099-023-00534-3

**Published:** 2023-02-24

**Authors:** Daniel Ekhlas, Juan M. Ortiz Sanjuán, Edgar G. Manzanilla, Finola C. Leonard, Héctor Argüello, Catherine M. Burgess

**Affiliations:** 1 Food Safety Department, Teagasc Food Research Centre, Ashtown, Dublin, Ireland; 2 School of Veterinary Medicine, University College Dublin, Dublin, Ireland; 3 Pig Development Department, Teagasc Moorepark, Fermoy, Co. Cork Ireland; 4 Grupo de Genómica Y Mejora Animal, Departamento de Genética, Facultad de Veterinaria, Universidad de Córdoba, Córdoba, Spain; 5 Animal Health Department, Veterinary Faculty, Universidad de León, León, Spain

**Keywords:** Pig farms, Antimicrobial prophylaxis, Zinc oxide, Antimicrobial resistance, Multi-drug resistance, *Escherichia coli*, Whole-genome sequencing

## Abstract

**Background:**

The prophylactic use of antimicrobials and zinc oxide (ZnO) in pig production was prohibited by the European Union in 2022 due to potential associations between antimicrobial and heavy metal usage with antimicrobial resistance (AMR) and concerns regarding environmental pollution. However, the effects of their usage on the bacterial AMR profiles on commercial pig farms are still not fully understood and previous studies examining the effect of ZnO have reported contrasting findings. The objective of this study was to examine the effects of antimicrobial and ZnO usage on AMR on commercial pig farms. Faecal and environmental samples were taken on 10 Irish commercial farms, of which 5 farms regularly used ZnO and antimicrobials (amoxicillin or sulphadiazine-trimethoprim) for the prevention of disease. The other 5 farms did not use ZnO or any other form of prophylaxis. *Escherichia coli* numbers were quantified from all samples using non-supplemented and supplemented Tryptone Bile X-glucuronide agar.

**Results:**

In total 351 isolates were phenotypically analysed, and the genomes of 44 AmpC/ESBL-producing *E. coli* isolates from 4 farms were characterised using whole-genome sequencing. Phenotypic analysis suggested higher numbers of multi-drug resistant (MDR) *E. coli* isolates on farms using prophylaxis. Furthermore, farms using prophylaxis were associated with higher numbers of isolates resistant to apramycin, trimethoprim, tetracycline, streptomycin, and chloramphenicol, while resistance to ciprofloxacin was more associated with farms not using any prophylaxis. Thirty-four of the 44 AmpC/ESBL-producing *E. coli* strains harboured the *bla*_CTX-M-1_ resistance gene and were multi drug resistant (MDR). Moreover, network analysis of plasmids and analysis of integrons showed that antimicrobial and biocide resistance genes were frequently co-located on mobile genetic elements, indicating the possibility for co-selection during antimicrobial or biocide usage as a contributor to AMR occurrence and persistence on farms.

**Conclusions:**

The results of this study showed evidence that antimicrobial and ZnO treatment of pigs post-weaning can favour the selection and development of AMR and MDR *E. coli*. Co-location of resistance genes on mobile genetic elements was observed. This study demonstrated the usefulness of phenotypic and genotypic detection of antimicrobial resistance by combining sequencing and microbiological methods.

**Supplementary Information:**

The online version contains supplementary material available at 10.1186/s13099-023-00534-3.

## Background

Antimicrobial resistance (AMR) is a global threat to human, animal, and environmental health. According to the One Health principles which were originally formulated in 2004 at the “One World, One Health” symposium in New York, transmission of disease and spread of AMR can only be prevented by acknowledging interconnections between humans, animals, and the environment [1]. Consequently, great efforts have been taken on a global scale to reduce the development and spread of AMR, especially in livestock production, where antimicrobials are extensively used, and have been associated with increased AMR in several studies [2]. Moreover, potential linkages of resistance to heavy metals and AMR due to cross-resistance, co-regulation, and co-occurrence of resistance genes on mobile genetic elements (MGEs) may select for persistence of AMR, even in the absence of antimicrobial-induced selective pressure on pig farms [3, 4].

*Escherichia coli* is a ubiquitous commensal for both humans and food-producing animals; however non-harmful strains co-exist with pathotypes which can cause gastrointestinal and urinary tract infections [5, 6]. In pig production, enterotoxigenic *E. coli* (ETEC) is one of the causative agents associated with post-weaning diarrhoea, which can result in ecological costs and impact negatively on farm profitability and animal health [7]. Therefore, antimicrobials and zinc oxide (ZnO) are commonly used for prophylactic treatment of piglets to prevent proliferation of ETEC during weaning [8]. However, in the European Union the prophylactic use of antimicrobials and therapeutic use of ZnO (2000–4000 ppm) has been banned due to possible associations with AMR development and spread, but also to reduce environmental pollution with heavy metals and antimicrobial residues via the use of manure in agriculture [9, 10]. Nevertheless, the impact of ZnO on AMR in livestock production is not yet fully understood.

The rise of MDR pathogenic *E. coli* in livestock is a major concern for animal, human, and environmental health [11]. In addition, MDR *E. coli* strains, or those carrying MGEs with resistance to last resort antimicrobials (e.g., *mcr-1* plasmid-encoded strains), constitute a risk to human health by their potential transmission to humans, mainly through the food chain [12]. Due to its relevance and the straightforward low-cost monitoring methods for *E. coli*, it is often used as a microbial indicator of AMR in farm studies [13].

Prophylactic use of antibiotics and/or ZnO at weaning has favoured the selection of MDR resistant strains, which usually carry MGEs. For instance, two studies by Bednorz, et al. [14] and Ciesinski, et al. [15] which examined *E. coli* isolates from pigs, observed associations between zinc supplementation and an increased number of MDR *E. coli*. As discussed by these two studies, one possible explanation for their findings could be the co-selection of *E. coli* strains carrying multiple ARGs and heavy metal resistance genes (MRGs), which are able to withstand zinc-induced selective pressure. Bednorz, et al. [14] reported an overall high diversity of *E. coli* in the porcine gut but indicated that this diversity can reduce during zinc administration, leaving primarily MDR *E. coli* strains [14]. Furthermore, ZnO also affects the host, inducing changes in intestinal morphology, immune system, hepatic proteome, mucins, and methylation, thus host-mediated selective pressures in response to ZnO treatment may additionally affect *E. coli* diversity in the porcine gut [16–19].

A number of studies have analysed the molecular mechanisms underlying the observed increase in AMR. In a study by Vahjen, et al. [20], increases in copy numbers of the *tet(A)* and *sul1* antimicrobial resistance genes (ARGs) were observed in response to high doses of dietary ZnO. These ARGs confer resistance to tetracyclines and sulphonamides respectively, suggesting that resistance to tetracycline or sulphonamides could potentially be increased with ZnO usage. This may occur because for instance, some efflux pumps that confer resistance to zinc can also confer cross-resistance to tetracycline and vice versa [20, 21].

Despite the information provided by these studies, most of them were small scale studies, performed under controlled conditions, and may not have captured the complexity of the commercial farm environment and other factors in pig production, such as the background resistance level due to the frequent use of antimicrobials or heavy metals on farms [22]. In addition, the new legislation which bans the prophylactic use of antibiotics and heavy metals to treat *E. coli* infections in pigs, creates a new window of opportunity to evaluate not only their link to AMR development, but also the potential impact of their removal on AMR [23, 24]. Consequently, the aim of this study was to investigate AMR *E. coli* diversity as a biomarker for monitoring the impact of the systematic use of antimicrobials and/or ZnO, or their removal, on AMR development on commercial pig farms.

## Results

### ***E. coli*** enumeration

**Fig. 1:**
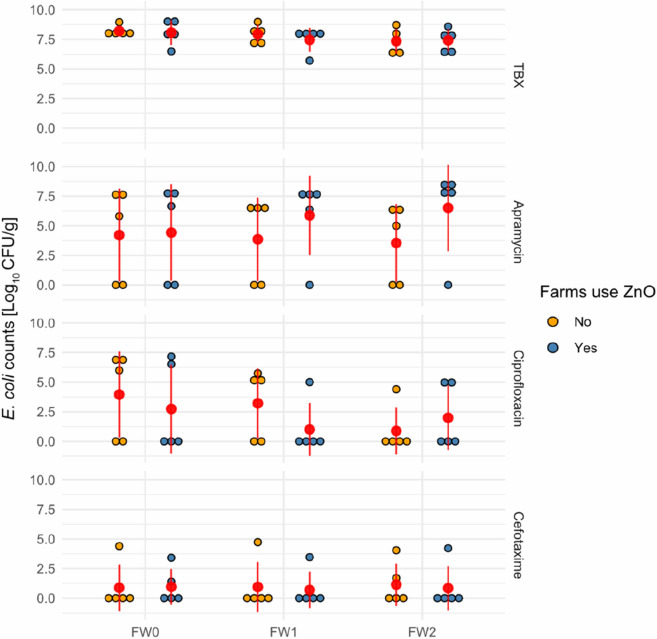
Enumeration of *E. coli* counts originating from faecal samples. *E. coli* counts from faecal samples from AB-ZnO using and AB-ZnO free farms using non-supplemented, apramycin- supplemented, ciprofloxacin-supplemented, and cefotaxime-supplemented TBX agar. Means and standard deviations are indicated in red. *E. coli* counts between AB-ZnO using and AB-ZnO free farms were compared per sample using the Mann-Witney U test (no significant differences were observed). FW0 = faecal sample at day of weaning; FW1 = faecal sample 1 week after weaning; FW2 = faecal sample 2 week after weaning

In the first part of this study, *E. coli* colony counts of faecal and swab samples were enumerated, using non-supplemented and antimicrobial supplemented Tryptone Bile X-glucuronide agar (TBX), a chromogenic agar for the detection and enumeration of *E. coli*. Overall no differences between *E. coli* concentrations on AB-ZnO farms and AB-ZnO free farms were observed, independent of the of the TBX media used. On non-supplemented TBX, *E. coli* counts reached mean values of 7.42–8.08 log_10_ CFU/g and 7.35–8.21 log_10_ CFU/g in faecal samples for AB-ZnO and AB-ZnO free farms, respectively (Fig. 1). Interestingly, differences between AB-ZnO and AB-ZnO free farms were observed when comparing *E. coli* concentrations derived from apramycin-supplemented plates. However, these differences did not reach significance (p > 0.05). At the day of weaning, faecal apramycin resistant *E. coli* numbers on AB-ZnO and AB-ZnO free farms were almost identical, with mean values of 4.43 log_10_ CFU/g and 4.22 log_10_ CFU/g, respectively. However, while the mean value of apramycin-resistant *E. coli* numbers decreased by 0.67 log_10_ CFU/g in AB-ZnO free farms after 2 weeks, on AB-ZnO farms there was an increase of 2.08 log_10_ CFU/g. Although differences between farms were not significant, apramycin-resistant *E. coli* numbers were slightly higher on AB-ZnO farms than AB-ZnO free farms.

For ciprofloxacin- and cefotaxime-resistant *E. coli* numbers no clear observations could be made, as these resistance types were infrequently observed on farms.

### Phenotypic antimicrobial resistance determination

After assessing phenotypic resistance profiles of 361 *E. coli* isolates which were taken from supplemented TBX agar, 351 isolates remained for further comparison (Table 1).

**Table 1:** Comparison of antimicrobial resistance patterns of *Escherichia coli* isolated on supplemented TBX agar plates

	*Apramycin-supplemented TBX*			*Ciprofloxacin-supplemented TBX*			*Cefotaxime-supplemented TBX*		
*Antimicrobial agent*	AB-ZnO free [n = 82] (%)	AB-ZnO [n = 127] (%)	p-value	AB-ZnO free [n = 46] (%)	AB-ZnO [n = 43] (%)	p-value	AB-ZnO free [n = 17] (%)	AB-ZnO [n = 36] (%)	p-value
*Apramycin*	100.00	100.00	–	26.09	23.26	0.757	35.29	97.22	< 0.001 (*)
*Trimethoprim*	63.41	86.61	< 0.001 (*)	78.26	90.70	0.107	64.71	66.67	0.888
*Nalidixic Acid*	18.29	11.02	0.138	76.09	83.72	0.370	41.18	19.44	0.094
*Tetracycline*	63.51	90.55	< 0.001 (*)	86.96	90.70	0.577	47.06	100.00	< 0.001 (*)
*Meropenem*	0.00	0.00	–	0.00	2.33	1.000	0.00	0.00	–
*Cefotaxime*	4.88	3.15	0.525	4.35	6.98	0.590	100.00	100.00	–
*Ciprofloxacin*	28.05	16.54	0.046 (*)	100.00	100.00	–	64.71	25.00	0.005 (*)
*Streptomycin*	69.51	92.13	< 0.001 (*)	84.78	69.77	0.090	35.29	91.67	< 0.001 (*)
*Chloramphenicol*	19.51	48.03	< 0.001 (*)	32.61	72.09	< 0.001 (*)	0.00	58.33	< 0.001 (*)
*Multi-drug resistant*	68.29	94.49	< 0.001 (*)	89.13	93.02	0.521	64.71	100.00	< 0.001 (*)

Results are divided based on the supplemented antimicrobial that was used and comparison between AB-ZnO using and AB-ZnO free farms was conducted using the Chi-square test. Statistical significance is indicated using an asterisk next to the p-value displayed. *N* number of resistant *E. coli*; multi-drug resistance was defined as resistant to at least three different antimicrobial classes

**Fig. 2:**
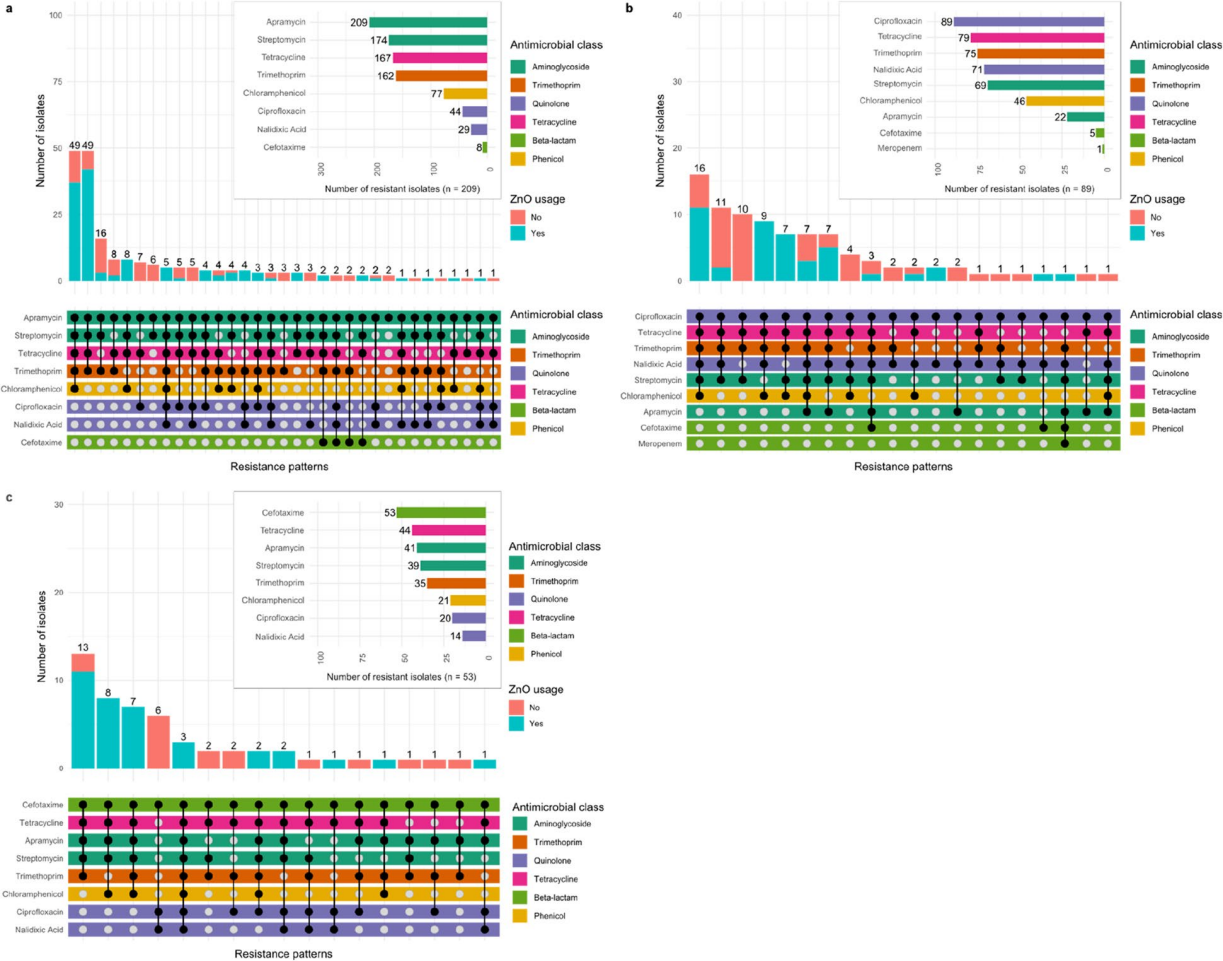
Phenotypic resistance patterns of 351 *Escherichia coli* isolates. Isolates were obtained from **a** apramycin-supplemented, **b** ciprofloxacin-supplemented, and **c** cefotaxime-supplemented TBX

When analysing the phenotypic resistance profiles of the 351 isolates, it was observed that certain resistance patterns occurred more frequently, independently of selective pressure, which was mediated by culturing isolates on supplemented TBX (Fig. 2). Moreover, it was observed that specific resistance profiles occurred more frequently on AB-ZnO or AB-ZnO free farms (Table 1). Based on the analysis of isolates derived from the apramycin-supplemented TBX, resistance to, trimethoprim (AB-ZnO free: 63.41%; AB-ZnO: 86.61%; p < 0.001), tetracycline (AB-ZnO free: 63.51%; AB-ZnO: 90.55%; p < 0.001), streptomycin (AB-ZnO free: 69.51%; AB-ZnO: 92.13%; p < 0.001), chloramphenicol (AB-ZnO free: 19.51%; AB-ZnO: 48.03%; p < 0.001), and multi-drug resistance (AB-ZnO free: 63.29%; AB-ZnO: 94.49%; p < 0.001) was observed more often in isolates originating from AB-ZnO using farms. Similar results were observed for isolates derived from cefotaxime-supplemented TBX, showing more frequently resistance to apramycin (AB-ZnO free: 35.29%; AB-ZnO: 97.22%; p < 0.001), tetracycline (AB-ZnO free: 47.06%; AB-ZnO: 100.00%; p < 0.001), streptomycin (AB-ZnO free: 35.29%; AB-ZnO: 91.67%; p < 0.001), chloramphenicol (AB-ZnO free: 0.00%; AB-ZnO: 58.33%; p < 0.001), and multi-drug resistance (AB-ZnO free: 64.71%; AB-ZnO: 100.00%; p < 0.001) on AB-ZnO farms. Isolates derived from ciprofloxacin-supplemented TBX, however showed only more frequent resistance to chloramphenicol (AB-ZnO free: 32.61%; AB-ZnO: 72.09%; p < 0.001) on AB-ZnO farms. Conversely, resistance to ciprofloxacin was higher in isolates originating from AB-ZnO free farms based on the analysis of isolates derived from apramycin-supplemented TBX (AB-ZnO free: 28.05%; AB-ZnO: 16.54%; p = 0.046) and cefotaxime-supplemented TBX (AB-ZnO free: 64.71%; AB-ZnO: 25.00%; p = 0.005) (Table 1).

Associations between AMR profiles were seen when visualizing phenotypic resistance patterns (Fig. 2). Specific patterns of co-occurrence of AMR were frequently observed. For instance, *E. coli* isolates showing phenotypic resistance to apramycin were also likely to be resistant to tetracycline, streptomycin, trimethoprim, and chloramphenicol (Fig. 2a, c).

Interestingly, the opposite was observed for *E. coli* isolates derived from the ciprofloxacin- supplemented plates. These isolates were less likely to be resistant to apramycin, but similar to the other isolates they commonly displayed phenotypic resistance to tetracycline, streptomycin, trimethoprim, and chloramphenicol, in addition to nalidixic acid resistance (Fig. 2b).

### Whole genome sequencing analysis of AmpC/ESBL *E. coli* isolates

In total, 64 AmpC β-lactamase-producing and ESBL *E. coli* isolates were confirmed by MALDI-TOF MS, of which 39 isolates derived from Farm8 (AB-ZnO farm), 21 isolates from Farm10 (AB-ZnO free farm) and 2 isolates from Farm2 (AB-ZnO free farm) and Farm6 (AB-ZnO farm), respectively. Interestingly, all of these isolates originated from farms that either used amoxicillin trihydrate together with ZnO or no treatment. No AmpC β-lactamase-producing and ESBL *E. coli* isolates were obtained from farms with sulphadiazine-trimethoprim together with ZnO treatment. Of the 64 isolates 44 (3 AmpC-positive and 41 ESBL positive) were selected for WGS, based on differences in their origin (farm, sample type, and selective plate), *E. coli* strain (identified via MALDI-TOF MS) and phenotypically expressed AMR.

Information on strain origin, serotypes, and sequence types, as well as ESBL genes present in all sequenced *E. coli* isolates are summarized in Additional file 1. The majority of β-lactamase resistance genes carried were the TEM-type gene *bla*_TEM-1B_1_, CTX-M-type gene *bla*_CTX-M-1_1_, and the OXA-type gene *bla*_OXA-1_1_.

No AmpC or ESBL resistance genes were detected for the phenotypically confirmed AmpC β-lactamase-producing isolates KB213, KB635, and KB643 which may be due to the set thresholds for minimum identity and coverage when screening for ARGs against the ResFinder database. The results of screening against the PointFinder database however, revealed mutations in the AmpC promotor associated with an AmpC-positive phenotype.

**Fig. 3:**
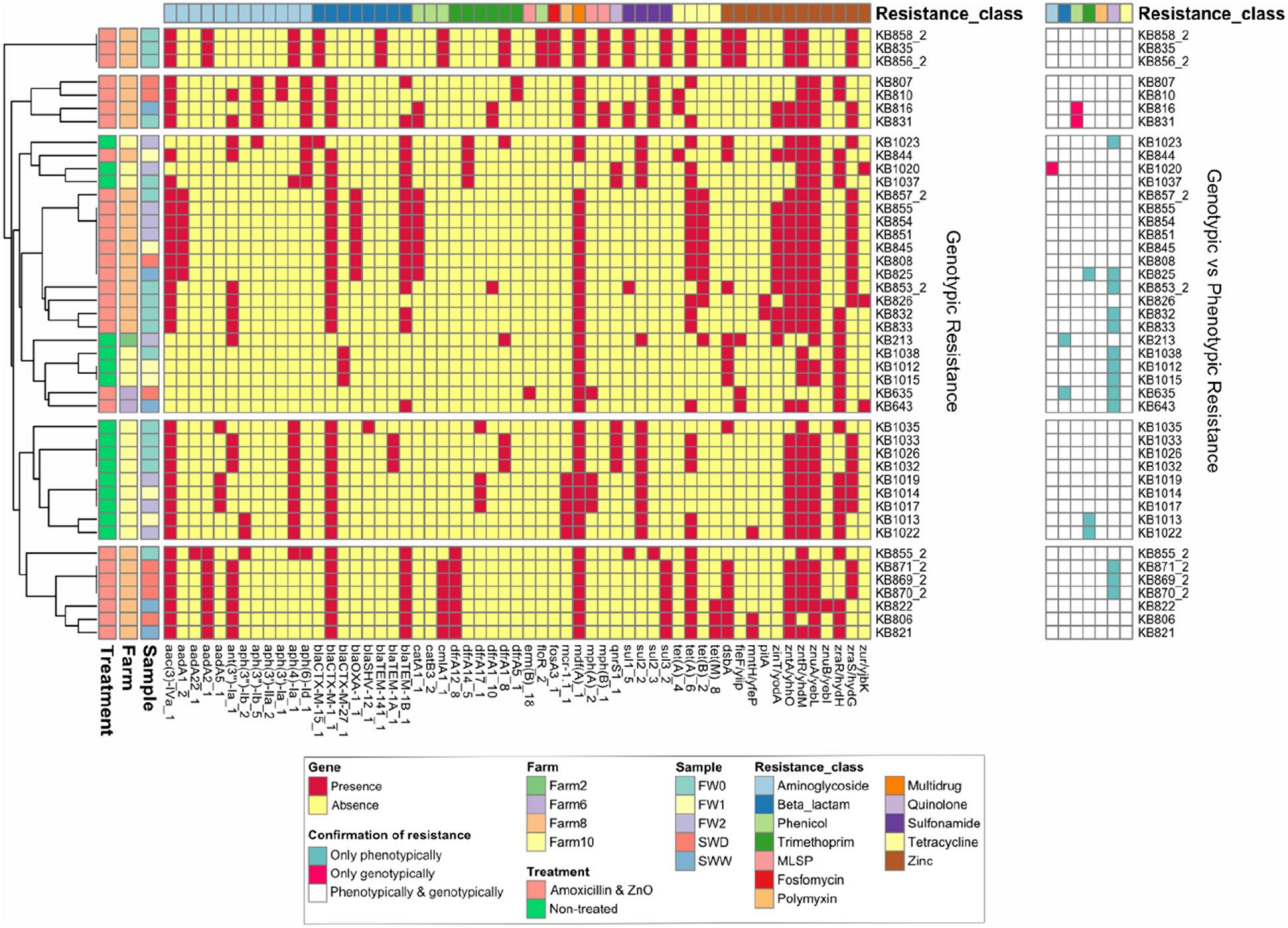
Identified resistance genes in AmpC / ESBL *E. coli* isolates. Genotypic and phenotypic results were additionally compared (see heatmap on the right). Sample abbreviations: *FW0* faecal sample at day of weaning, *FW1* faecal sample 1 week after weaning, *FW2* faecal sample 2 week after weaning, *SWD* environmental swab sample of cleaned drinkers and feeders before contact with piglets, *SWW* environmental swab sample of cleaned walls and floors before contact with piglets

Apart from this, further analysis of ARGs showed that ARGs such as *tet(A)* (confers resistance to tetracycline), *mdf(A)* (confers multi-drug resistance), and *aac(3)-IV* (confers resistance to apramycin) were commonly detected in the *E. coli* isolates (Fig. 3). In total, twelve different MRGs were identified conferring resistance to zinc. All isolates carried at least one zinc resistance gene.

Interestingly, the colistin resistance gene *mcr-1.1* was detected in five isolates from an AB-ZnO free farm. Phenotypic expression of colistin resistance was confirmed for all five strains (≥ 16 µg/mL) using Vitek2^®^ and the AST-GN96 susceptibility test card, which tested for polymyxin B. In most cases, phenotypic and genotypic AMR profiles of *E. coli* isolates matched, except for resistance to quinolones which often could only be confirmed phenotypically.

AB-ZnO and AB-ZnO free farms clustered in different branches in the Euclidean distance analyses performed based on ARGs in the samples (Fig. 3). These clusters however, seemed to be less impacted by the sample type when compared to farm and ZnO and antimicrobial usage. Although distinct clusters were formed, no difference in the frequency of ARGs between AB-ZnO farms and AB-ZnO free farms was observed.

**Fig. 4:**
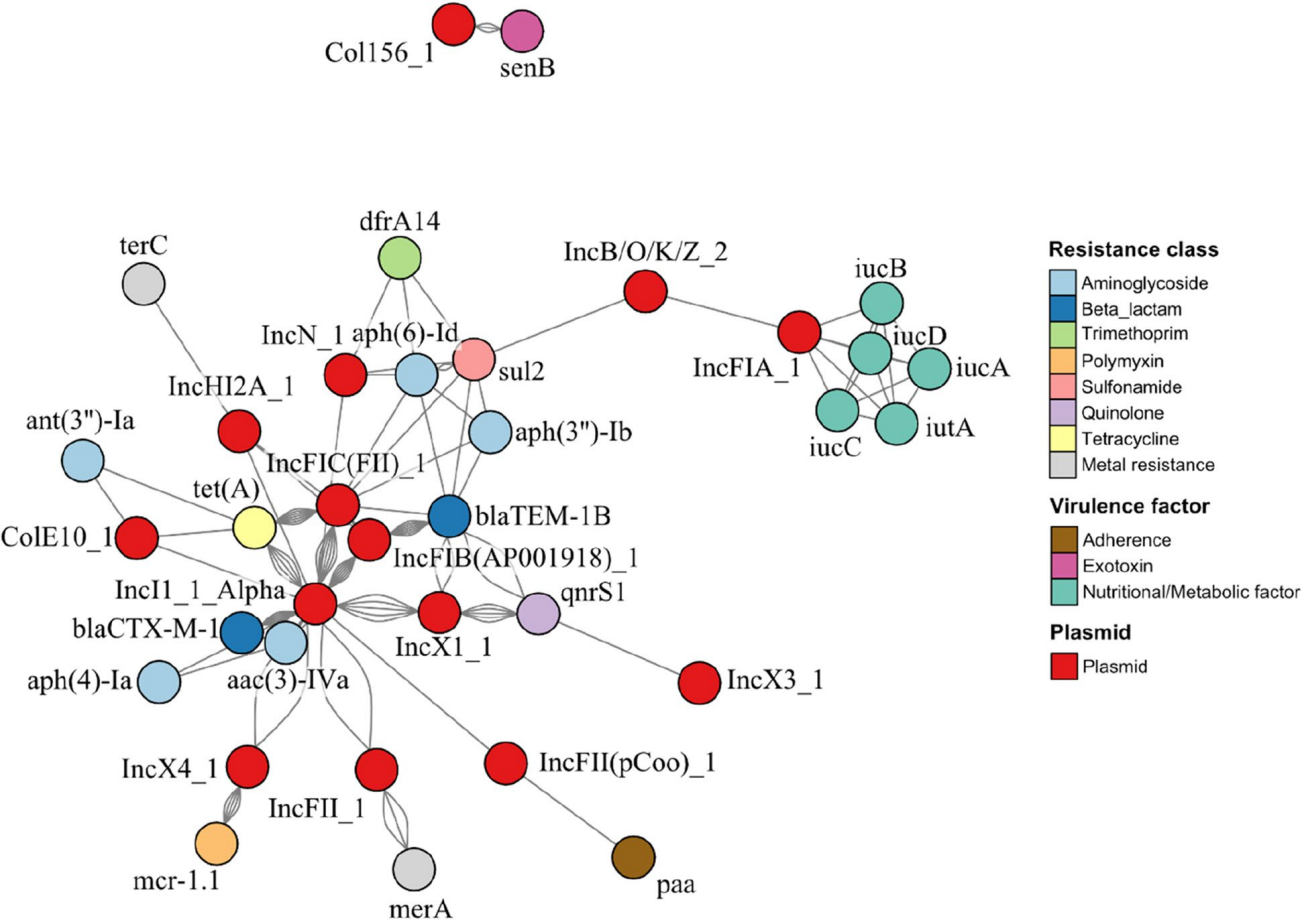
Network analysis of identified plasmids, resistance genes, and virulence genes of AmpC/ESBL *E. coli* isolates. Each connection between plasmids and genes indicates that the gene was found on the plasmid. Connections between genes indicate their co-occurrence on the same plasmid per isolate. Connections between plasmids indicate their co-occurrence in the same isolate. Node placement is based on the Fruchterman-Reingold algorithm

To further analyse which resistance and virulence genes were carried by plasmids, a network analysis was conducted. Based on the network analysis, IncI1_1_Alpha and IncFIB (AP001918) were the main carriers of *bla*_CTX-M-1_ and *bla*_TEM-1B_ respectively (Fig. 4). Additionally, *aac(3)-IV* was frequently co-located together with *bla*_CTX-M-1_ on contigs identified as IncI1_1_Alpha. Similarly, *tet(A)* was frequently found on IncI1_1_Alpha and IncFIC(FII), but rarely together with other resistance genes. Overall, ARGs conferring resistance to aminoglycosides were predominantly found on plasmids. Other ARGs conferring resistance to trimethoprim, sulfonamide, tetracycline, and polymyxin were also identified, often together with ARGs of another resistance classes. The colistin resistance gene *mcr-1.1* was only found on the IncX4 plasmid in five isolates, which did not carry any other virulence or resistance genes. Only two MRGs were found on plasmids, namely *terC* (confers resistance to tellurium) on IncH2A_1 and *merA* (confers resistance to mercury) on IncFII_1. Virulence factors on plasmids included an aerobactin-producing *iucABCD-iutA* operon located on the IncFIA_1 plasmid which was carried by a single isolate from Farm2 (KB213), the *senB* encoding for the *Shigella* enterotoxin 2 found in three isolates from Farm10 (KB1012, KB1015, and KB1038) carried by the Col156 plasmid, and the gene *paa*, encoding for adherence factors was only found in an isolate from Farm8 (KB835). All virulence factors identified were not co-located with other virulence factors or resistance genes. Virulence factors were only found in isolates originating from faecal samples.

In total, 30 unique integrons carrying ARGs were identified. Of these integrons, 21 were identified in isolates from Farm8, eight in isolates from Farm10, and one in the isolate from Farm2. Twenty-two integrons were found in isolates from faecal samples, while eight were found in isolates from environmental swab samples. Considering that three isolates which originated from faeces on Farm8 carried two AMR integrons, it can be summarized that of 19 of 31 isolates (61.29%) from faecal samples and 8 of 13 isolates (61.54%) from environmental samples carried at least one AMR integron. Overall, the ARGs *aadA* (confers aminoglycoside resistance), *dfrA* (confers trimethoprim resistance), *cmlA* (confers chloramphenicol resistance), and *qacF* (confers resistance to quaternary ammonium compounds) were predominant on all integrons. Additionally, the ARG *ant(3’’)-Ia* (confers aminoglycoside resistance) was also found on two integrons.

**Fig. 5:**
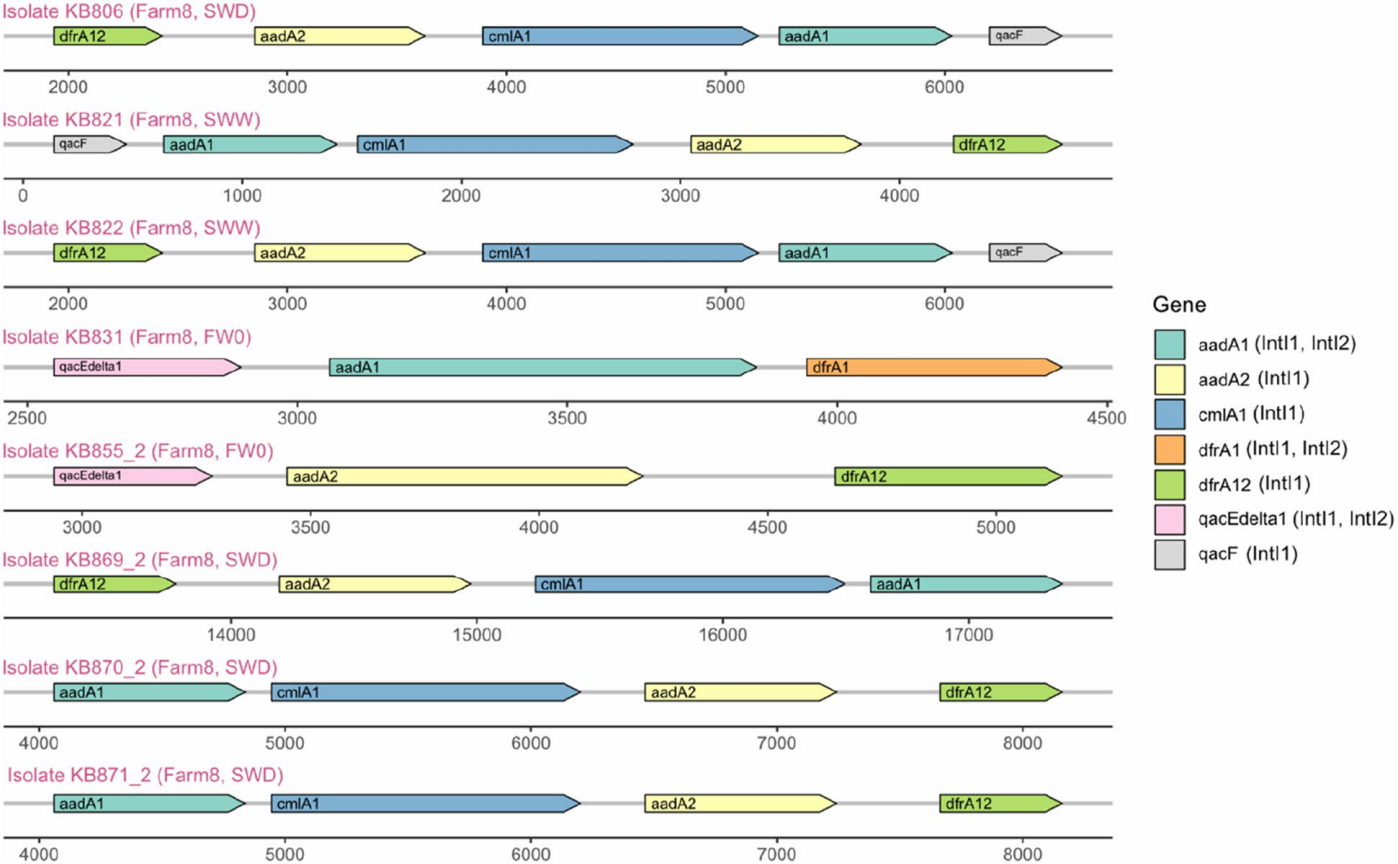
Integrons with at least three ARGs, MRGs, or BRGs found in all samples. Potential integron classes are written in brackets for each gene in accordance with entries in the INTEGRALL database. Sample abbreviations: *FW0* faecal sample at day of weaning, *SWD* environmental swab sample of cleaned drinkers and feeders in empty pens, *SWW* environmental swab sample of cleaned walls and floors in empty pens

Eight of the AMR integrons carried more than three resistance genes, with the aminoglycoside ARGs *aadA1* and *aadA2*, the chloramphenicol ARG *cmlA1*, and the trimethoprim ARG *dfrA12* being predominant (Fig. 5).

**Fig. 6:**
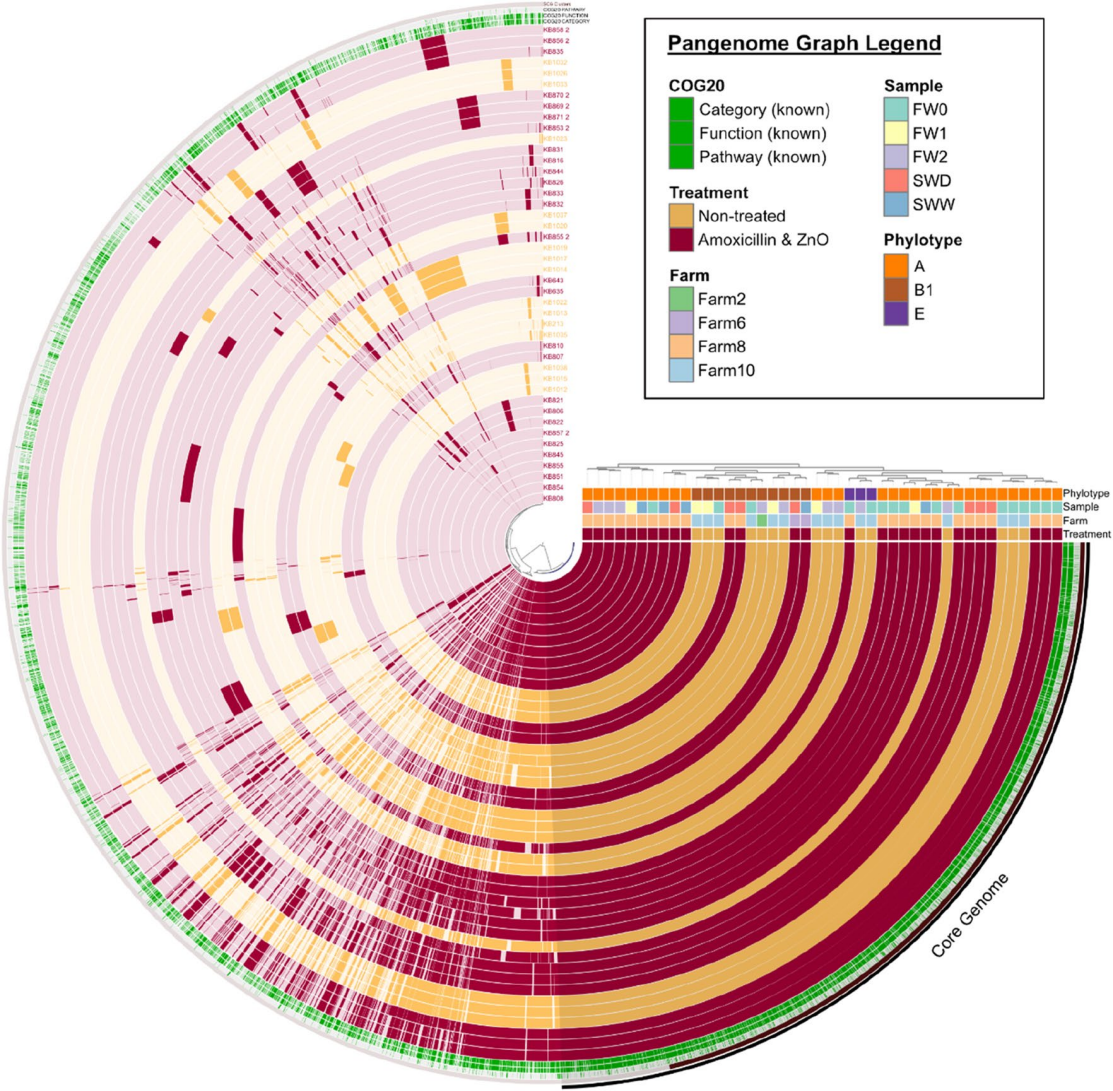
Pangenome analysis for 44 AmpC / ESBL *E. coli *isolates. Gene clusters are indicated as opaque coloured elements and ordered based on their presence and absence, using the Euclidean distance and Ward clustering (see also inner dendrogram). Samples are hierarchically clustered based on the frequency of gene clusters (see also outer dendrogram). Sample abbreviations: *FW0* faecal sample at day of weaning, *FW1* faecal sample 1 week after weaning, *FW2* faecal sample 2 week after weaning, *SWD* environmental swab sample of cleaned drinkers and feeders in empty pens, *SWW* environmental swab sample of cleaned walls and floors in empty pens

All sequenced AmpC / ESBL *E. coli* isolates were compared using a pangenome analysis approach (Fig. 6). The pangenomic analysis resulted in 9,901 gene clusters with 218,672 genes, in the 44 *E. coli* genomes, which were identified using NCBI blastp. The core genome of all isolates consisted of 3,229 gene clusters with 147,907 genes. Additionally, Euclidean and Ward clustering resulted in several clusters independent of sample type. An apparent association of clusters with phylotypes of the *E. coli* isolates was observed. Phylotype A (30 isolates; 23 from AB-ZnO farms & 7 from AB-ZnO free farms) was predominantly identified, followed by B1 (11 isolates; 4 from AB-ZnO farms & 7 from AB-ZnO free farms) and E (3 isolates; 1 from a AB-ZnO farm & 2 from AB-ZnO free farms).

## Discussion

In this study, *E. coli* isolates derived from 10 Irish pig farms, 5 farms that regularly used either amoxicillin or sulphadiazine-trimethoprim, together with ZnO, for 2-weeks as prophylactic treatment during weaning, and 5 farms that did not use any prophylaxis during weaning, were compared for the presence of AMR *E. coli*.

*E. coli* counts were first compared between farms by culturing samples on non-supplemented and antimicrobial-supplemented TBX agar. *E. coli* counts obtained from non-supplemented TBX were almost identical between AB-ZnO farms and AB-ZnO free farms. Furthermore, no decrease in total *E. coli* counts in faecal samples was observed 2 weeks post-weaning. Similar results were observed in a study of Ciesinski, et al. [15], who also did not report any differences in *E. coli* counts in faeces of ZnO-treated and non-treated pigs. When comparing *E. coli* counts obtained from apramycin-containing TBX, numerical differences between faecal samples originating from AB-ZnO farms and AB-ZnO free farms were observed from 1 week post-weaning. However, these differences were not statistically different, possibly due to the limited number of farms and samples taken. Therefore, larger number of farms and samples may be necessary in future studies to further investigate these differences. As discussed by Fairbrother, et al. [8] and Broom, et al. [25], administration of ZnO-supplemented feed may not result in an overall reduction of *E. coli*, but may rather result in a shift of the diverse *E. coli* community to more resistant strains, as reported by Ghazisaeedi, et al. [26] and Bednorz, et al. [14]. Comparison of AMR profiles of *E. coli* isolates confirmed the observations made by Ghazisaeedi, et al. [26] and Bednorz, et al. [14], showing a higher frequency of *E. coli* isolates resistant to apramycin, trimethoprim, tetracycline, streptomycin, chloramphenicol, and with multi-drug resistance on AB-ZnO farms. Interestingly, resistance to ciprofloxacin was more frequently observed for isolates originating from AB-ZnO free farms, although the reasons for this were not clear.

When analysing phenotypic resistance profiles of the 351 isolates, it was observed that certain resistance patterns occurred more frequently, independent of the selective pressure mediated by culturing isolates on supplemented TBX. Commonly observed resistance patterns across all supplemented agars included phenotypic resistance to tetracycline, streptomycin, trimethoprim, and chloramphenicol. Similar resistance patterns were observed by Ciesinski, et al. [15], who investigated the effects of zinc oxide (2103 mg/kg diet) use on MDR *E. coli* in the porcine gut. In their study, they described three frequently observed resistance patterns: (1) Ampicillin-Streptomycin-Tetracycline; (2) Streptomycin-Sulfamethoxazole/Trimethoprim-Tetracycline; and (3) Ampicillin-Streptomycin-Sulfamethoxazole/Trimethoprim-Tetracycline. Although they used different antimicrobials for phenotypic AMR determination, the observed resistance patterns were similar.

These results may indicate a selective adaptive response of *E. coli* to treatment on farms using sulphadiazine-trimethoprim and ZnO, but also non-selective adaption possibly due to cross-resistance or co-location of ARGs on MGEs, as previously reported by a study of Rovira, et al. [27]. To further investigate possible co-selection events, 44 AmpC/ESBL *E. coli* isolates were sequenced using a whole-genome sequencing approach.

Analysis of resistance profiles of AmpC / ESBL *E. coli* isolates showed that the ARGs *aac(3)-IV* (N-acetyltransferase AAC(3)-IV) and *tet(A)* (efflux pump) were two of the most frequently observed ARGs in all AmpC / ESBL *E. coli* isolates. Both of these ARGs were previously reported to be frequently found on plasmids [28, 29]. This was also confirmed by the network analysis in the present study, showing the frequent occurrence of *tet(A)* on IncI1_1_Alpha and IncFIC(FII). The ARG *aac(3)-IV* was also frequently found on IncI1_1_Alpha and was commonly co-located with *bla*_CTX-M-1_ on the same plasmids, carried by the majority of the AmpC / ESBL *E. coli* isolates. The occurrence of IncI1_1_Alpha*-bla*_CTX-M-1_ plasmids in livestock production has been regularly reported in Europe and is of great concern for public health [30–32]. These plasmids are highly transmissible between Enterobacteriaceae, including *Salmonella enterica* serovar Typhimurium, and have been reported in food-producing animals but also in clinical settings, with evidence that this ARG may disseminate via *E. coli* contamination in the food chain [33–36]. A study by Hiley, et al. [36] reported the occurrence of the plasmid IncI1 in *S.* Typhimurium isolates associated with a food poisoning outbreak in 2015. Similarly, *bla*_CTX-M-1_ has been reported in a *Klebsiella pneumoniae* outbreak in Spain within an intensive care unit by Mena and colleagues [37]. However, to the best of the author’s knowledge, no study yet reported a clinical disease outbreak caused by Enterobacteriaceae carrying IncI1_1_Alpha*-bla*_CTX-M-1_.

On Farm10 five *mcr-1.1*-positive ESBL *E. coli* isolates were obtained which showed phenotypic resistance to polymyxin B. Network analysis revealed that *mcr-1.1* was located on the plasmid IncX4, which has been reported in previous clinical and environmental studies [38–41]. To the best of the authors’ knowledge this is the first study reporting *mcr-1.1*-positive *E. coli* isolates on Irish commercial pig farms. Co-occurrence of the IncX4-*mcr-1.1* plasmid with the IncI1_1_Alpha plasmid in the same bacterial isolate was observed for two isolates.

Integron analysis also showed frequent co-occurrence of ARGs such as *dfrA*, *aadA, cmlA,* and *qacF* conferring trimethoprim, aminoglycoside, chloramphenicol, and resistance to quaternary ammonium compounds respectively. These ARGs were reported before on pig farms by Mencía-Ares, et al. [42], mainly associated with class 1 integrons and may partially explain the frequent occurrence of the previously discussed resistance patterns. The majority of these integrons were found in isolates from Farm8. Thus, the use of antimicrobials such as amoxicillin (β-lactam) and sulphadiazine-trimethoprim (sulphonamide and trimethoprim) may facilitate the co-selection of ARGs conferring resistance to these agents, with ARGs conferring resistance to aminoglycoside such as apramycin and streptomycin, chloramphenicol, quaternary ammonium compounds, and tetracyclines. Although the number of isolates may not allow clear conclusions to be drawn, co-selection and co-location of ARGs of different antimicrobial classes on MGEs may explain the increased phenotypic resistance observed to these antimicrobial classes, including multi-drug resistance. Furthermore, carriage of multiple ARG-plasmids may additionally allow the persistence of AMR isolates, such as *mcr-1.1*-positive *E. coli* strains, on farms due to co-selection and fitness advantages against other niche competitors during antimicrobial and ZnO usage.

Apart from that, it was observed that all sequenced *E. coli* strains carried at least one MRG conferring resistance to zinc. No zinc-resistance genes were found on MGEs and it remains unclear in the current study settings, if ZnO plays a crucial role in comparison to AMU in terms of AMR selection or the observation of the increased numbers of MDR *E. coli* observed on AB-ZnO farms. Ghazisaeedi, et al. [26] and Bednorz, et al. [14] reported increases in MDR *E. coli* associated with ZnO supplementation. While Bednorz, et al. [14] concluded in their study that increases in MDR *E. coli* could be due to co-selection under selective pressure and enhanced bacterial conjugation rates [43, 44], Ghazisaeedi, et al. [26] suggested that co-selection may not allow higher tolerance to zinc, but MDR isolates may be more resistant to environmental stresses such as zinc exposure per se.

Phenotypic differences between farms were also reflected in the genotypic results, although phenotypically observed resistance could not always be confirmed genotypically. Mutations in *gyrA* (encodes DNA gyrase) and *parC* (encodes DNA topoisomerase IV) often confer resistance to quinolones, as well as *qnr* or *aac(6')-Ib-cr* resistance genes. These mutations are not included in the ResFinder database, which would explain why resistance to quinolones was frequently confirmed phenotypically, but not genotypically in many isolates [45]. However, additional screening of these 13 isolates, showing phenotypic resistance to quinolones, against the PointFinder database confirmed mutations in *gyrA* (in 12 cases), *gyrA* and *parC* (in 4 cases), and *gyrA, parC,* and *parE* (in 2 cases). Moreover, the three isolates which were confirmed phenotypically as AmpC β-lactamase-producers, but for which no AmpC β-lactamase encoding resistance gene was found, could be further confirmed using the PointFinder database.

Distinct clusters of *E. coli* isolates from AB-ZnO and AB-ZnO free farms were observed in the ARG heatmap and the pangenomic analysis. Clusters in the ARG heatmap were more associated with differences in ARG carriage, while clusters in the pangenome analysis seemed to be more associated with *E. coli* phylotypes. The predominant phylotype observed on all pig farms was phylotype A, and phylotype A and B1 in the case of AB-ZnO free farms. These phylogenetic groups have been reported before in faecal samples of pigs and are known to represent mainly commensal, but also intestinal, pathogenic strains [46]. Additionally, three strains belonged to phylogenetic group E, of which *E. coli* O157:H7 is a well-known member [47, 48]. Two of these three strains originated from Farm10 and belonged to the serotype O167:H4. The other strain serotype could not be identified. The serogroup O167 has been reported before as enterotoxigenic or enteroinvasive *E. coli*. Virulence factors associated with these pathogroups were also found on MGEs, such as *paa* and *senB*, however not in the same isolates.

The results of the pangenome analysis showed that the core genome of all sequenced isolates consisted of 3,229 gene clusters, while the total pangenome consisted of 9,901 gene clusters. This is in accordance with results of Cummins, et al. [49], who reported that pangenomes vary between *E. coli* sequence types. Thus, they reported that *E. coli* core genome sizes can range from 3,066 to 4,058 clusters, with a pangenome size range from 6,825 to 27,634 gene clusters.

Future studies in this field should include in-depth analysis methods such as long-read sequencing or combined hybrid approaches, which with current costs, do not permit sequencing the number of isolates included in this study. As shown in this study, the information gained by using short-sequencing methods is sufficient to identify potential pathogens, their plasmids, and resistance genes, and demonstrates the benefits of combining culture-dependent and culture-independent analyses to provide a more complete picture.

## Conclusions

In conclusion, the results of this study showed prophylactic use of antimicrobials and ZnO for 14 days post-weaning on farms favoured the selection and development of AMR and MDR *E. coli*. Frequently observed AMR patterns and higher prevalence of AMRs on AB-ZnO farms could be partially explained by co-location of ARGs on MGEs such as integrons or plasmids, which are potentially more frequently transferred under ZnO/antimicrobial exposure. The study also identified the first *mcr-1-*encoding *E. coli* isolated in Irish pig production. This study demonstrated the usefulness of combining microbiological and sequencing methods to evaluate the impact of antimicrobial and heavy metal removal on AMR on pig farms.

## Methods

### Farm sampling

Samples were collected from 10 pig farms in Ireland between September 2020 and April 2021. Five farms regularly used prophylactic treatments with antimicrobials and ZnO for the first 2 weeks post-weaning (AB-ZnO farms) for more than 3 years and five farms did not use any such medication for the 3 years prior to sample collection (AB-ZnO free farms). Of the AB-ZnO farms Farm4, Farm6, and Farm8 used amoxicillin trihydrate (Stabox [15 mg/kg], Virbac, France) and Farm1 and Farm3 used sulphadiazine-trimethoprim (Sulfoprim 15% [15 mg/kg], Univet Limited, Ireland), all in combination with ZnO (3000 ppm, i.e. 3 g ZnO/kg feed). A total of five different samples were taken on each farm, including two environmental and three faecal samples. Piglets on each farm were weaned at an age of approximately 28 days and allocated to nursery pens. Immediately before entry of piglets to the pens, environmental samples of cleaned walls, floors, feeders, and drinkers were taken using a sponge-stick soaked in 10 mL neutralizing buffer (3 M^™^, Ireland). One sponge was used to swab an area of 1 m^2^ of cleaned walls and floors, and another sponge was used to swab cleaned drinkers and feeders. Freshly voided faecal droppings of piglets were collected on the day of weaning (day 0), as well as on days 7 and 14 post-weaning. Three faecal droppings per batch were pooled together in a sterile plastic container and mixed with a sterile micro-spatula. All samples were transported to the laboratory on ice in a polystyrene cooling box and processed within 24 h.

### ***E. coli*** isolation

Faecal samples were mixed with a sterile micro-spatula before processing in the laboratory. From each sample, 4 g of faeces were transferred into a sterile 150 mL container and mixed with 36 mL maximum recovery diluent (MRD, Oxoid, United Kingdom) to obtain a 1:10 dilution. After vortexing, the suspended faecal samples were transferred into filter stomacher bags (Grade products, United Kingdom) and were homogenized for 60 s with a Star Blender LB 400 (VWR, Ireland).

For the swab samples, 20 mL sterile Tryptone Soy Broth (TSB, Oxoid, United Kingdom) was added directly to the swab bag and the sponge was squeezed several times. Swabs were incubated for 1 h at 37 °C for bacterial enrichment.

Faecal and environmental sample suspensions were serially diluted and plated in duplicate on non-supplemented and supplemented Tryptone Bile X-glucuronide agar (TBX, Merck KGaA, Germany) plates containing either no antibiotics or 16 mg/L apramycin sulfate salt (Sigma-Aldrich, Germany, according to Yates, et al. [50], 1 mg/L ciprofloxacin (Thermo Scientific, Ireland, or 4 mg/L cefotaxime sodium salt (Sigma-Aldrich, Germany), in accordance with the Clinical and Laboratory Standards Institute; CLSI M100-ED32:2022). Antimicrobials were dissolved in sterilized deionised water and filtered using sterile 0.22 µm syringe filters (GVS, USA), before addition to media. After samples were plated they were incubated for 24 h at 44 °C. It is important to mention that enumeration of colony counts after incubation was only conducted for faecal samples, due to the enrichment step for the environmental samples. Up to 5 colonies per sample were selected and transferred into cryogenic tubes (Technical Service Consultant Ltd., UK), which were stored at −80 °C.

### Phenotypic antimicrobial resistance determination

In total, 361 isolates were analysed using the Kirby-Bauer disc diffusion susceptibility test [51]. Isolates were streaked out on Tryptone Soya Agar (TSA, Oxoid, United Kingdom) and incubated for 24 h at 37 °C. Following this, one colony of each isolate was transferred to 10 mL TSB and incubated for 16 h at 37 °C to prepare overnight cultures (ONCs). ONCs were serial diluted in MRD. Suspensions were spread on Mueller–Hinton Agar (MHA, Sigma-Aldrich, Germany) followed by the application of antibiotic discs on the plates. Nine antimicrobials were tested, including apramycin (15 µg), trimethoprim (5 µg), nalidixic acid (30 µg), tetracycline (30 µg), meropenem (10 µg), cefotaxime (5 µg), ciprofloxacin (5 µg), streptomycin (10 µg), and chloramphenicol (30 µg) (Oxoid, UK). The MHA plates were then incubated for 24 h at 37 °C. Susceptibility tests were conducted in duplicate and *E. coli* strain ATCC^®^ 25922 was used as a reference. Inhibition zones were measured and evaluated in accordance with clinical breakpoints published by the CLSI and the European Committee on Antimicrobial Susceptibility Testing (EUCAST). The susceptibility test results of 10 of 361 isolates were not consistent with the results of the selective plating of the faecal and environmental samples and were excluded from further analysis, thus 351 isolates remained. Where a colistin resistance gene was detected via WGS analysis, phenotypic expression of polymyxin B resistance was assessed using the Vitek2^®^ and the AST-GN96 susceptibility test card (BioMérieux, United Kingdom).

### Identification and confirmation of AmpC/ESBL *E. coli*

Isolates showing phenotypic resistance to cefotaxime were screened for AmpC and extended-spectrum β-lactamases (ESBLs) using the MASTDISCS^®^ AmpC ESBL detection set (Product code: D68C, Mast Group Ltd., UK).

Isolates that were confirmed as ESBL and AmpC β-lactamase producers were analysed via MALDI-TOF MS (Matrix-Assisted Laser Desorption Ionization-Time of Flight Mass Spectrometry) at DAFM Laboratories Backweston (Department of Agriculture, Food and the Marine, Ireland) to confirm that they were *E. coli*. Briefly, AmpC and ESBL isolates were streaked out on TSA and incubated at 37 °C for 24 h. One colony of each isolate was transferred into 2 mL screw cap micro tubes and suspended in 300 µl ultrapure water (18.2 MΩxcm). Subsequently, 900 µl pure ethanol (Merck, Ireland) were added and vortexed. The suspensions were centrifuged at 13,000 rpm for 2 min, the supernatant removed, and the pellet allowed to dry for 5–10 min. Pellets were re-suspended in 25 µl of 70% formic acid (Merck, Ireland) before 25 µl acetonitrile (Sigma-Aldrich, Ireland) was added. The suspensions were centrifuged at the same settings as previously. For each isolate, 1 µl of supernatant was transferred onto different spots of a MALDI target plate. After the spots dried, 1 µl of a-Cyano-4-hydroxycinnamic acid matrix solution (HCCA, Bruker Daltronics GmbH, Germany) was added. After another drying step, isolates were analysed via MALDI-TOF using the Bruker MALDI Biotyper IVD (version 10) and the FlexControl software (version 3.4) (Bruker Daltronics GmbH, Germany). The US IVD Bacterial Test Standard (BTS, Bruker Daltronics GmbH, Germany) was used for validation and as a quality control for the MALDI-TOF analysis. Isolates were analysed using the ‘MBT_AutoX’ AutoXecute acquisition method with a mass-to-charge ratio (m/z) range of 1.06–20.14 kDA. Obtained protein spectra were aligned against the BDAL Bruker database. Logarithmic alignment scores and consistencies were calculated by the MBT Compass Explorer software (version 4.1; Build 100), ranging from 0.00 to 3.00 and A(+ + +)−C(−) respectively. Only results with a score greater than 2.00 and a consistency of A were accepted, resulting in 64 confirmed *E. coli* isolates, of which 44 isolates were selected based on their origin, taxonomy, and resistance profiles for further analysis via whole-genome sequencing.

### Whole genome sequencing analysis of AmpC/ESBL *E. coli* isolates

DNA of confirmed AmpC / ESBL *E. coli* isolates was extracted using the DNeasy^®^ UltraClean^®^ Microbial kit (Qiagen, Germany). DNA extracts were quantified using the Nanodrop^TM^ 1000 Spectrophotometer and the Qubit 4.0 Fluorometer (Invitrogen, ThermoFisher Scientific, United Kingdom). DNA libraries of extracts were prepared using the Illumina DNA prep kit in accordance with the manufacturer’s instructions, followed by 2 × 150 bp sequencing with P2 reagent on the Illumina Nextseq2000 platform. Obtained raw reads were cleaned using Trimmomatic (v0.38) [52] and the quality of trimmed raw reads was assessed using FastQC (v0.11.8) and MultiQC (v1.9) [53, 54]. The taxonomic assignment of *E. coli* strains was confirmed using Kraken 2 (v2.1.1) and the Kraken 2 PlusPF database (last update: 2021-May-17). Subsequently, reads were assembled to scaffolds using SPAdes (v3.15.3) and the –isolate tag [55]. QUAST and MultiQC were used to assess the quality of assembled scaffolds [53, 56]. Afterwards, scaffolds were examined for antimicrobial resistance genes and heavy metal resistance genes via ABRicate (v1.0.1; https://github.com/tseemann/abricate) using the ResFinder database (last update: 2021-Mar-27) with –minid 80 –mincov 75, and Bacmet-Scan (v1.0) with default settings and the BacMet2 database containing experimentally confirmed resistance genes [57]. Additionally, scaffolds were screened against the PointFinder database (last update: 2022-Apr-22) with a minimum threshold for identity of 70% and a minimum length of 60% via the CGE online platform (Center for Genomic Epidemiology; http://www.genomicepidemiology.org/) to account for resistance caused by point mutations [58]. *E. coli* serotypes were determined using ABRicate and the EcOH database (last update: 2021-Mar-27) with the same identity and coverage thresholds. Multi-locus sequence typing was conducted using mlst (v2.18.0, https://github.com/tseemann/mlst) [59]. To further examine for MGEs and their association to specific ARGs and MRGs, assembled scaffolds were separated into chromosomal and plasmidic scaffolds using Platon (v1.6) [60]. Plasmids based on plasmidic scaffolds were identified using PlasmidFinder (last update: 2021-Mar-27) and were analysed for ARGs, BRGs and MRGs, and virulence factors using ABRicate together with the ResFinder and VFDB database (last update: 2021-Mar-27) with the previous mentioned thresholds, as well as Bacmet-Scan (v1.0). Only plasmidic scaffolds which were identified by PlasmidFinder and carried resistance or virulence genes were included in the subsequent network analysis. Scaffolds obtained by SPAdes were screened for integrons using IntegronFinder (v2.0) [61]. Resistance genes on integrons were identified using ABRicate, BacMet2, and the mobilome analysis pipeline created by José F. Cobo-Díaz (https://github.com/JoseCoboDiaz/ARG-contig_mobilome_analysis). The pangenome of all 44 isolates was reconstructed using anvi’o (v7.1).

### Data analysis and visualization

All graphs except for the pangenome analysis were visualized using R v4.0.2 (2020-06-22) and RStudio v2021.09.2 [62]. *E. coli* concentrations were visualized using the ggplot2 package [63] and were compared using the Mann-Witney U test via the rstatix package (v0.7.0) [64]. In cases where no colonies were obtained at the lowest dilution, a value of 0 log_10_(CFU/mL) was set, representing 1 cfu in an undiluted sample. Upset plots for visualizing phenotypic resistance patterns of *E. coli* isolates (Fig. 2) were done using the ComplexUpset package [65]. Statistical comparison of phenotypic resistance patterns was performed by conducting the Chi-square test via Minitab 17 (v17.1.0). Heatmaps showing the presence and absence of ARGs of AmpC/ESBL *E. coli* isolates were visualized using the pheatmap package [66]. Network analysis of plasmids, virulence factors, and resistance genes was conducted using the igraph package (v1.3.0) and the Fruchterman-Reingold algorithm for node placement [67, 68]. Integrons were visualized using the gggenes package (v0.4.1) [69]. The pangenome analysis of the 44 AmpC/ESBL *E. coli* isolates was performed using anvi’o (v7.1) in accordance with the workflow for microbial pangenomics (https://merenlab.org/2016/11/08/pangenomics-v2/) [70]. Briefly, assembled scaffold.fasta files were first converted into anvi’o contig databases using the ‘anvi-gen-contigs-database’ program. Afterwards, genes in scaffolds were identified by using Prodigal to screen for open reading frames, which were further annotated using the NCBI’s Clusters of Orthologous Groups database (‘anvi-run-ncbi-cogs’ program) [71, 72]. Genes were annotated against four provided HMM profiles of anvi’o using hidden Markov models (‘anvi-run-hmms’ program). The pangenome was computed by (1) calculating the amino acid sequence similarities and comparison across all genomes via NCBI blastp, (2) eliminating weak matches between amino acid sequences using minbit heuristics of 0.5 [73], and (3) identifying clusters using the MCL algorithm [74] (‘anvi-pan-genome’ program). The pangenome was visualized and edited using the anvi’o interactive interface and the program ‘anvi-display-pan’. Figures were arranged and modified using Affinity Designer (v1.8.5.703, Serif).

## Supplementary Information

**Additional file 1. **Summary of analysed AmpC / ESBL *E. coli* isolates obtained in this study. Sample abbreviations: FW0 = faecal sample at day of weaning; FW1 = faecal sample 1 week after weaning; FW2 = faecal sample 2 week after weaning; SWD = environmental swab sample of cleaned drinkers and feeders before interaction with piglets; SWW = environmental swab sample of cleaned walls and floors before interaction with piglets.

## Data Availability

Raw sequences from the whole-genome sequencing analysis of the AmpC / ESBL *E. coli* isolates are publicly accessible under BioProject: PRJNA901700.
